# User Identification across Asynchronous Mobility Trajectories

**DOI:** 10.3390/s19092102

**Published:** 2019-05-07

**Authors:** Mengjun Qi, Zhongyuan Wang, Zheng He, Zhenfeng Shao

**Affiliations:** 1 National Engineering Research Center for Multimedia Software, School of Computer Science, Wuhan University, Wuhan 430072, China; 2017282110542@whu.edu.cn (M.Q.); hezheng@whu.edu.cn (Z.H.); shaozhenfeng@whu.edu.cn (Z.S.); 2 Shenzhen Research Institute of Wuhan University, Shenzhen 518057, China; 3 School of Remote Sensing and Information Engineering, Wuhan University, Wuhan 430072, China

**Keywords:** GPS (Global Positioning System) trajectory, identification resolution, frequent pattern, similarity measure

## Abstract

With the popularity of location-based services and applications, a large amount of mobility data has been generated. Identification through mobile trajectory information, especially asynchronous trajectory data has raised great concerns in social security prevention and control. This paper advocates an identification resolution method based on the most frequently distributed TOP-N (the most frequently distributed N regions regarding user trajectories) regions regarding user trajectories. This method first finds TOP-N regions whose trajectory points are most frequently distributed to reduce the computational complexity. Based on this, we discuss three methods of trajectory similarity metrics for matching tracks belonging to the same user in two datasets. We conducted extensive experiments on two real GPS trajectory datasets GeoLife and Cabspotting and comprehensively discussed the experimental results. Experimentally, our method is substantially effective and efficiency for user identification.

## 1. Introduction

Position services [1], including GPS and positioning of cellular communication networks, have become widespread. Multi-source mobility trajectory datasets generated by mobile phones, networks, and vehicle navigation, contain rich information about human activities. More and more researchers are working on application based on location information, including socio-economic development and wellbeing [2], human individual mobility characteristics [3,4], traffic analysis [5,6,7], travel guides [8,9,10,11], user behavior modelling [12], cross domain recommendation [13], identifying and predicting lifestyles [14], construction for 3D geospatial data [15],and so on.

In real-world applications, trajectory data is typically generated by a multi-source platform, or data is temporally separated. An identification algorithm is very necessary to link the trajectory information of users at different time periods, which focuses on improving the quantity and quality of mobility data. In addition, multi-source data based identification algorithms have been extensively studied, but there are few studies specifically for asynchronous trajectory information.

In this paper, we focus on the problem of user identification based on asynchronous mobility trajectory data. In other words, it matches the trajectories from two different periods to determine whether two users’ trajectories belong to the same person. The study that determines the user to which the trajectory belongs by measuring the similarity between the two trajectories has potential application value in social security prevention. A typical application scenario is that criminals often use multiple mobile phones in turn. Without the real name system of mobile phones, it is impossible to know that these mobile phones (e.g., 2) are from the same person. When we acquire the trajectory data of the mobile phone by mobile base station or GPS positioning, if the trajectories of the two phones (we do not know the ownership in advance) are highly similar, they are likely generated by different phones of the same user. In this way, we can judge that these phones are held by the same user.

It needs to be explained that the asynchronous trajectory means two trajectories do not coincide at all time. Due to the fact that the human behavior always changes with time, there is a certain inconsistency in the mobility trajectory, which thus results in difficulties for asynchronous identification resolution. In order to facilitate the description of the method, we use the following symbols to describe the problem. The trajectory of an object can be defined as the chronological sequence as follows:

(1) $$T=\{id,\left(LO_{1},LA_{1},t_{1}\right),\cdots ,\left(LO_{k},LA_{k},t_{k}\right)\}$$

Among them, the id is the person’s identity, which is a positive integer. Each position point is represented by a three-dimensional coordinate$\left(LO_{k},LA_{k},t_{k}\right)$. $LO_{k}$ and $LA_{k}$, respectively, represent the longitude and latitude of the k-th recorded point of the track. $t_{k}$ denotes the recording time.

Two sets of trajectories of multiple objects at two different periods are described as

(2) $$S=\{T_{id}^{s}|id=1,2,\cdots ,m\}$$

(3) $$Q=\{T_{id}^{q}|id=1,2,\cdots ,n\}$$

The problem to be solved is to match the trajectory in *S* with the trajectory in *Q*, provided that each trajectory in *S* has at least one trajectory in *Q* belonging to the same object. This is not a simple problem because the trajectories in *S* and *Q* do not have any overlap in time, and we only use the trajectory information of two objects to calculate the similarity without exploiting any other information. We determine the trajectory most similar to the target as the matching result.

This paper proposes a framework called TOP-N (which uses TOP-N most frequently distributed regions) to achieve mobility identification resolution. In addition, a similarity method based on probabilistic deviation, angle cosine and weighted Jaccard similarity is used to measure similarity of two trajectories. The location where the the trajectory points are frequently distributed is usually more identifiable. For example, users usually stay in residences, companies, etc. for a long time; that is, track points are frequently distributed in these areas. For the areas that the user routinely passes every day, even if the speed is very fast, but as long as the time span is large enough, they are still the areas where the track points are frequently distributed. The infrequently distributed areas are those that the user randomly visits, which have no user identity implication. So, our method first finds TOP-N most frequently distributed regions of the trajectory, called TOP-N region set. As for the set of TOP-N regions, we calculate the scalar product of all pairs of co-distributed regions and then compute the product of the moduli of two TOP-N region sets, taking the quotient of the two as its similarity, namely, cosine similarity. Obviously, the more common regions that the two trajectory distributions share, the greater their similarity. However, there is an alternative possibility. More precisely, the number of co-distributed areas in trajectories *T* and $T_{1}$ is *n* while the number of non-coinciding areas is $m_{1}$; the number of co-distributed areas of *T* and $T_{2}$ is also *n* while the number of non-co-distributed areas is $m_{2}$. Assuming that $m_{1}$ > $m_{2}$, it is very likely that $T_{2}$ is more similar to *T* than $T_{1}$. Therefore, both the co-distributed and the non-co-distributed areas must be accounted for. In another situation, there are three trajectories, and their co-distribution region’s number and non-co-distributed region’s number are the same. In this case, if we want to distinguish which two trajectories are more similar, we need to consider the frequency values of the trajectory points distributed in each region. In short, when calculating the similarity between two trajectories, two sequences need to be compared and reordered to find the pairs of co-distributed regions.

The main contributions are summarized as follows.

1. An user identification resolution method based on TOP-N frequent distribution regions of asynchronous mobility trajectory is proposed.
2. The effectiveness of TOP-N method was verified on two real mobility trajectory datasets.
3. We prove that in the identification task of asynchronous trajectory, the length in time of the trajectory has a great influence on the identification accuracy.

## 2. Related Work

Our work in this paper is closely related to the topic of the user identification and similarity search based on human mobility data. In recent years, some researchers studied identity resolution in cyberspace [1,16,17,18,19,20], while [21,22,23,24,25,26,27] exploited identity identification through trajectory information. In [25,26,28,29,30] studied the uniqueness of the trajectories of different objects. Ref. [25] particularly showed that it is enough to identify users in multiple trajectories based on this uniqueness. Because the behaviors and habits of the same person are generally periodic, their activity trajectories also show a certain periodicity [30]. For example, [14] proved that sometimes, some people’s expected lifestyles can be analysed by the trajectory periodicity.

The identification task based on mobility information is roughly divided into two scenarios of synchronization and asynchronous, and the synchronization scenario generally refers to cross-platform trajectory matching. In [26], a robust distance function called EDWP (Edit Distance with Projections) is proposed to match the trajectory data at inconsistent and variable sampling rates by dynamic interpolation. Ref. [23] proposed a hotspot-matrix method, using cosine of the distribution matrix of two trajectories to express the similarity. This method can achieve a certain degree of recognition accuracy, but the amount of calculation is huge. Ref. [24] introduced a Signal-Jaccard co-filtering method for matching multi-source trajectory, which can also be used to identify asynchronous trajectory. Only considering the same distribution areas, [22] calculates cosine of all pairs in the same regions and uses learning-to-rank to improve the identification. Particularly, a inverted-index strategy is used to reduce the computational cost. Although the computational complexity can be substantially reduced, it needs to find all uniform regions from a large number of regions of two trajectories. Ref. [31] presented a concept to capture both spatial and temporal diversity aspects of the linkage, called K-L diversity. In [32], based on space-time matching points, a real-time trajectory similarity measure method was proposed. Similarly, in [21], the authors proposed to achieve identification by time-space matching trajectory points. The matching points of the two trajectories require that the points are close in both time and space. This method is only for synchronous scenarios that is unavailable in our problem.In a related work, [27] recently proposed a method called CDTraj2vec for user trajectory matching across social networks. This method uses PV-DM (Distributed Memory Model of Paragraph) model and paragraph2vec algorithm to extract the positional access order features in the trajectory sequence, which obtains the vector representation of the user trajectory and thus represents the trajectory similarity by the vector. However, this method ignores the distribution frequency of track points and the important information of track transition status.

## 3. Proposed Method

We propose an effective method to deal with user identification across asynchronous mobility trajectories. Our method includes the following procedures: (1) Find out the TOP-N areas where the trajectories of the objects are most frequently distributed; (2) The similarity is represented by the cosine, probability deviation and weighted Jaccard similarity of TOP-N region sets; (3) Determine the trajectory most similar to the target as the matching result.

### 3.1. Finding Top-N Regions

In order to find out the TOP-N regions where the trajectory points are most frequently distributed, we take into account the case that the rectangle area is determined by the highest and lowest longitude and latitude of the city. For the sake of simplified calculation, we ignore the location points exceeding the latitude and longitude of the city coverage.

#### 3.1.1. Latitude and Longitude Grid

The city is regarded as a rectangular region enclosed by the highest and the lowest longitude and latitude. The rectangular region is divided into M×N small grids. Each small grid corresponds to a geographical area of the city. Different divisions of the area will affect the amount of computation and identification accuracy.

#### 3.1.2. Distribution Matrix of A Trajectory

We count the frequency of the appearance of track points in each small area and then map them to a M×N dimensional matrix to obtain the distribution of the track of an object. The specific method for calculating the distribution matrix is presented as the following steps.

First, we suppose the observation scope is within the area represented by the following formula:

(4) L=[max(LO),min(LO)]×[max(LA),min(LA)]

Here, min(LO), max(LO), min(LA), max(LA) indicate the minimum and maximum values of the city’s longitude and latitude, respectively. Accordingly, we mesh the entire area into many small rectangle grids with a height of h_lo and a width of h_la. The total number of grids is thus M×N. We use the expression

(5) $$L_{M\times N}=\left\{l_{ij}\right\}\left(1\leq i\leq M,1\leq i\leq N\right)$$

to represent the entire city’s latitude and longitude network, where

(6) $$M=\frac{max(LO)-min(LO)}{h\_lo},N=\frac{max(LA)-min(LA)}{h\_la}$$

Assume that the element corresponding to the *i*-th row and the *j*-th column of the two-dimensional matrix $A_{M\times N}$ is $a_{ij}$, the value is determined by the frequency that the trajectory appears in the *i*-th row and the *j*-th column region of the latitude and longitude network, where

(7) $$i=ceil\left(\frac{LO-min(LO)}{h\_lo}\right),j=ceil\left(\frac{LA-min(LA)}{h\_la}\right)$$

We use the same method to individually count the frequency of the trajectory in each small area by adopting the one-to-one mapping rule.

#### 3.1.3. Top-N Region Set

A threshold PN is first set and we filter out all elements in the distribution matrix that are larger than PN (or areas whose trajectory distribution frequency is greater than the value) to form a set called region_list. The region_list is ranked in frequency and the TOP-N elements with the highest frequency values are used to produce the TOP-N region set. A TOP-N region set is regarded as

(8) $$TN_{id}=\left\{\left(a_{ij}^{k},\left(i,j\right)\right)k=1,2,\cdots ,TOP-N\right\}$$

where $a_{ij}^{k}$ denotes the distribution frequency of the trajectory in the *k*-th region and (i,j) represents the coordinate of this region. In order to avoid the computational overflow, uniform standardization is applied to all values of TOP-N region set. Namely, the TOP-N region set`s element value of each trajectory is divided by the total number of points of the trajectory. The algorithm pseudo code is summarized in Algorithm 1.

**Algorithm 1:** Get TOP−N region sequence from trajectory *T*.

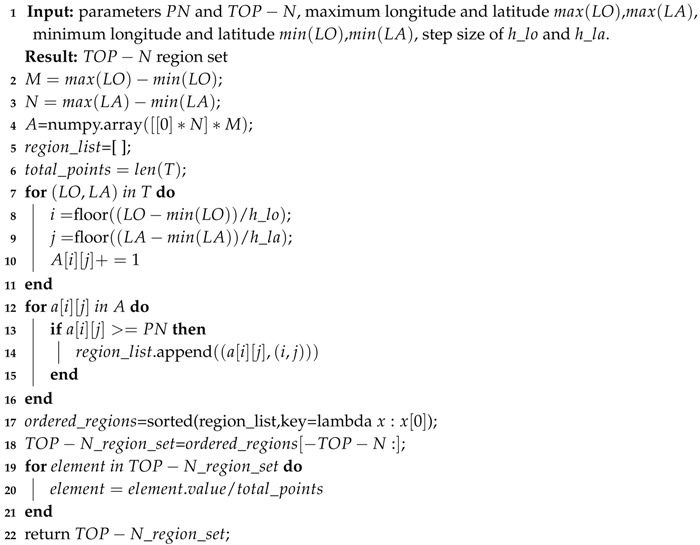



### 3.2. Similarity of Top-N Region Set

The task of calculating the similarity of two trajectories is converted into calculating the similarity of two TOP-N region sets. There are many ways to measure the similarity between two sets. Hereby, we discuss three methods for comparing similarities. The first one is angle cosine of set, which is similar to the angle cosine of vector, but the order of the set must be adjusted first. The second one is the probability deviation, which explores the probability deviation of two trajectories appearing in all co-distributed areas. The more close the probability of trajectories appearing in the same areas, the more similar they are. Last one is weighted Jaccard similarity which was first proposed by Ioffe et al. [33].

#### 3.2.1. Angle Cosine of $TN_{1}$ and $TN_{2}$

Step 1: Find all common regions of the TOP-N sequences $TN_{1}$ and $TN_{2}$. We use the following notation to express them.

(9) $$BD_{TN_{1},TN_{2}}=\left\{\left(a_{ij}^{TN_{1}},a_{mn}^{TN_{2}}\right)|m=i,n=j\right\}$$

$\left(a_{ij}^{TN_{1}},a_{mn}^{TN_{2}}\right)$ indicate the value pairs which fall between 0 and 1, and they are distributed in the same area (i,j).

Step 2: First, calculate the modular product of TOP-N sequences $TN_{1}$ and $TN_{2}$. And then, calculate their vector product of the co-distributed regions.

(10) $$H=\sum_{\left(a_{ij}^{TN_{1}},a_{mn}^{TN_{2}}\right)\in BD_{TN_{1},TN_{2}}}a_{ij}^{TN_{1}}\cdot a_{mn}^{TN_{2}}$$

(11) $$G={\left(\sum_{a_{ij}^{k}\in TN_{1}}{\left(a_{ij}^{k}\right)}^{2}\right)}^{\frac{1}{2}}\cdot {\left(\sum_{a_{mn}^{k}\in TN_{2}}{\left(a_{mn}^{k}\right)}^{2}\right)}^{\frac{1}{2}}$$

Note that (10) and (11) do not calculate the same object, while the set $BD_{TN_{1},TN_{2}}$ in (10) is the intersection of $TN_{1}$ and $TN_{2}$ in (11). Equation (10) represents the vector product of $BD_{TN_{1},TN_{2}}$, and (11) denotes the modular product of the TOP−N region sets $TN_{1}$ and $TN_{2}$. It is seen that the co-distributed and the non-co-distributed areas are both considered.

Step 3: Formulate the similarity between TOP-N sequences $TN_{1}$ and $TN_{2}$, namely,

(12) $$sim_{TN_{1},TN_{2}}=|BD_{TN_{1},TN_{2}}|\cdot \frac{H}{G}$$

H and G are given by (10) and (11) respectively, with $|BD_{TN_{1},TN_{2}}|$ being the number of co-distributed regions of $TN_{1}$ and $TN_{2}$.

#### 3.2.2. Probability Deviation of $TN_{1}$ and $TN_{2}$

In order to simplify the model, this paper uses the frequency to represent the probability, i.e., the ratio of the trajectory points in each region to the total number of trajectory points. Similar to angle cosine method, the probability deviation similarity need to find out all regions which both $TN_{1}$ and $TN_{2}$ contain, so as to accumulate the probability deviation of each co-distributed area and to calculate the average deviation. The smaller the probability deviation is, the greater the similarity is. We also consider the number of co-distributed areas, and the larger this value is, the more similar the trajectories are.

(13) $$\begin{array}{c} S=\left.\sum_{\left(P_{ij}^{TN_{1}},p_{mn}^{TN_{2}}\right)\in BD_{TN_{1},TN_{2}}}{\left(P_{ij}^{TN_{1}}-P_{mn}^{TN_{2}}\right)}^{2}\right.\\ \left.+\frac{1}{2}\cdot (\sum_{P_{ij}^{TN_{1}}\in TN_{1}\wedge P_{ij}^{TN_{1}}\notin BD_{TN_{1},TN_{2}}}{\left(P_{ij}^{TN_{1}}\right)}^{2}\right.\\ \left.+\sum_{P_{mn}^{TN_{2}}\in TN_{2}\wedge P_{mn}^{TN_{2}}\notin BD_{TN_{1},TN_{2}}}{\left(P_{mn}^{TN_{2}}\right)}^{2}\right. \end{array}$$

(14) $$sim_{TN_{1},TN_{2}}=e^{\frac{|BD_{TN_{1},TN_{2}}|}{|TN_{1}|+|TN_{2}|}}\cdot e^{-\frac{S}{|TN_{1}|+|TN_{2}|}}$$

$P_{ij}^{TN_{1}}$ indicates the probability that $T_{1}$ appears in the area (i,j). It should be noted that the method also deals with the non-coincidence regions. Assuming that the probability of the trajectory $T_{1}$ appearing in the region *r* is *P*, $T_{2}$ does not appear in *r* with the probability is 0. The probability deviation of $T_{1}$ with $T_{2}$ appearing in the region *r* is P−0=P. Similarly, the number of areas where $T_{1}$ and $T_{2}$ co-distributed is also considered. In fact, the comparison of the similarity of two TOP−N sequences does not require that they have the same length.

#### 3.2.3. Weighted Jaccard Similarity of $TN_{1}$ and $TN_{2}$

The weighted Jaccard similarity measures the resemblance between two weighted sets. In our problem, TN refers to a collection of TOP-N region sets. The weight of each region is the corresponding visiting frequency. The similarity of $TN_{1}$ and $TN_{2}$ is defined as

(15) $$WJS=\frac{\sum min(a_{ij}^{TN_{1}},a_{mn}^{TN_{2}})}{\sum max(a_{ij}^{TN_{1}},a_{mn}^{TN_{2}})}$$

(16) $$sim_{TN_{1},TN_{2}}=|BD_{TN_{1},TN_{2}}|\cdot WJS$$

(16) computes the weighted Jaccard similarity of $TN_{1}$ and $TN_{2}$. ∣BD∣ indicates the number of elements in the set BD.

## 4. Experimental Evaluation

### 4.1. Description of Datasets

The experiments in this paper are conducted on two real datasets, including GeoLife [34] and CabSpotting [35]. For GeoLife dataset, we only use the latitude and longitude of each recorded point, while the time information associated with each point in CabSpotting dataset is used. In addition, in order to facilitate the verification of the identification accuracy, trajectories need to be tagged as their id. The labels are encoded by a series of numbers, which are used to check whether the result of matching is correct or not after the identification.

GeoLife is a GPS-recorded dataset that records the trajectory information from 182 users from April 2007 to August 2012, collected by Microsoft Research Asia. This dataset records the wide-ranging outdoor activities of the tested users, including both routines for going home and going to work and some entertainment and sports activities such as shopping, sightseeing, dining, hiking and cycling. The trajectories were recorded by different GPS loggers and GPS phones, and have a variety of sampling rates. 91.5% of the trajectories are logged in a dense representation, e.g., every 1–5 s or every 5–10 m per point. Trajectories of 112 users are roughly located in Beijing, and, thus, the latitude and longitude mostly span between 39.5–41 and 115.4–117.6, respectively. For the sake of calculation, it needs to delete all points whose latitude and longitude are out of this range. Furthermore, in order to retain more experimental data, we also keep the trajectory points in Beijing more than 30%, so a total of 164 user trajectories are used for experiments. As most of the points in the trajectory are too time-intensive (every 1–5 s per point), one point is taken by every four points in experiments. As a result, we can reduce the computational cost without affecting the identification accuracy.

Cabspotting dataset is a public GPS dataset that contains trace information of 536 taxis in San Francisco for less than a month (17 May 2008–10 June 2008). This dataset has been recently used by Piorkowski et al. [36] to show that certain macroscopic characteristics specific to clustered mobile wireless networks are prevalent in real mobility traces. Due to the inherent characteristics of this dataset(such as common routes of taxis) and the fact that the trajectories are spatially constrained to lie on the streets, the points are less unique. Because the tracks contain a lot of noise points, we delete the high-speed moving point ahead, for instance, the points in which the adjacent speed exceeds 20 m/s.

### 4.2. Experimental Method

Each trajectory in GeoLife and Cabspotting datasets is divided into two segments, so that two verification sets are obtained respectively. Particularly, the GeoLife dataset is split into $D_{g_{1}}$ and $D_{g_{2}}$, and Cabspotting dataset is split into $D_{c_{1}}$ and $D_{c_{2}}$. During the experiments, each trajectory in $D_{g_{2}}$ and $D_{c_{2}}$ is divided into 15 equal parts. We then take 9 copies in order (1–9, 6–15) one time, so that every test trajectory can include 2 sub-trajectories for testing. For $D_{g_{1}}$ and $D_{g_{2}}$, 164×2 times of identification experiments are performed with 164 trajectories while 536×2 identification experiments are performed with 536 trajectories for $D_{c_{1}}$ and $D_{c_{2}}$. The ratio of successful identification times to total identification times is taken as the identification accuracy.

Prior to recognition, all training trajectories and test trajectories are used to acquire their TOP-N sequences. Thus, calculating the similarity of two trajectories only needs to compare their TOP-N sequences. It notes that the recognition accuracy is related to the selection of parameters, including the size of steps h_lo and h_la of the latitude and longitude as well as the number of TOP-N areas where the trajectories are most frequently distributed. In addition, the time length of the trajectory also affects the identification accuracy. In experiments, our TOP-N method includes three variants by embedding different similarity metrics, denoted by TOP-N-PRO, TOP-N-COS and TOP-N-WJS, respectively.

### 4.3. Experimental Results and Analysis

#### 4.3.1. Effects of h_lo and h_la

We have before explained that h_lo and h_la determine the size of the small mesh area after the partition. Here the effect of h_lo and h_la on the experimental results is discussed briefly. The side length of the small grid is taken as 50–500 m. As a result, the corresponding value of h_lo is 0–0.006 and the value of h_la is 0–0.005.

**Figure 1:**
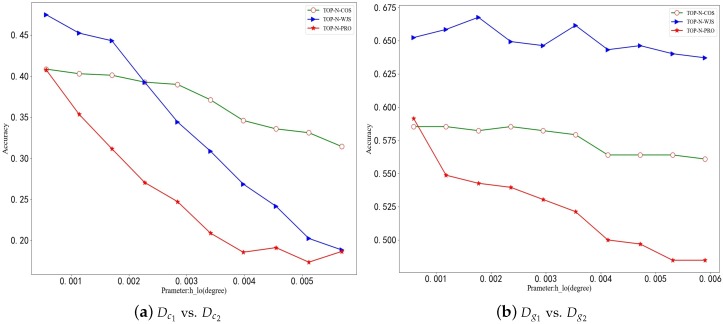
Comparing TOP-N-COS, TOP-N-PRO and TOP-N-WJS by accuracy vs. h_lo. (**a**) Cabspotting, (**b**) Geolife.

Experimental results on two datasets (GeoLife and Cabspotting) are shown in Figure 1. As shown in Figure 1, as the longitude h_lo increases, the identification accuracy tends to decrease. The smaller the h_lo is, the more precise the actual position of each grid region becomes. We thus can easily distinguish the actual distributed locations of the user`s trajectories. Conversely, the larger the h_lo is, the less accurate the locations are. As a result, one grid is likely to correspond to a number of completely different physical locations. On the other hand, when h_lo is too small, it is possible to divide one physical location into multiple grids, thus resulting in inaccurate matching. In addition, the h_lo accuracy graph exhibits obvious oscillating situation. The fluctuation may be due to the division rule on urban areas. As mentioned before, if one real area is divided by different h_lo, there will be multiple situations. In a simple example, two different users (*a* and *b*) often pass by the same large park, where the user A passes the street on the west side of the park and the user B passes through the small road on the east side. When the division by h_lo is small, the east and west of the park are separated into two grids. When the division of h_lo is large, they will fall into the same grid. Obviously, the division of the latter makes two users’ trajectories more similar due to the increase in their same distribution areas.

#### 4.3.2. Effects of Top-N

**Figure 2:**
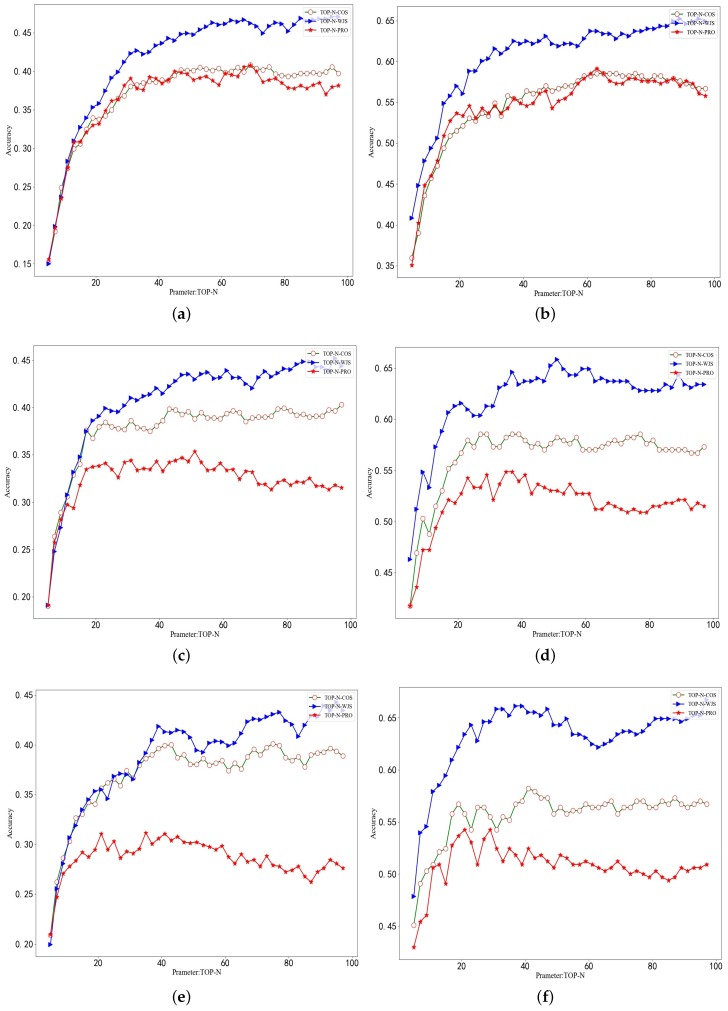
Comparing TOP-N-COS, TOP-N-PRO and TOP-WJS by accuracy vs. TOP-N. (**a**,**c**,**e**) $D_{c_{1}}$ vs. $D_{c_{2}}$, (**b**,**d**,**f**) $D_{g_{1}}$ vs. $D_{g_{2}}$.

In order to discuss the effect of TOP-N on the identification accuracy, we should control the values of h_lo and h_la. The experimental results from Section 4.3.1 show that as the longitude h_lo increases, the recognition accuracy tends to decrease. Therefore, three specific h_lo and h_la parameter values are selected, namely h_lo=0.000578 and h_la=0.000450, h_lo=0.001137 and h_la=0.0009, h_lo=0.001705 and h_la=0.001350, respectively, which accordingly correspond to a small grid side length of 50 m, 100 m, 150 m. We set the TOP-N value from 5 to 100 and performed an experimental analysis on $D_{g_{1}}$ vs. $D_{g_{2}}$ and $D_{c_{1}}$ vs. $D_{c_{2}}$. Figure 2 shows comparisons among probability deviation method, weighted Jaccard similarity and cosine method. In Figure 2a,b we reveal the experimental results with h_lo=0.000578, h_la=0.000450, (c) and (d) show the experimental results of h_lo=0.001137, h_la=0.0009, and (e) and (f) takes h_lo=0.001705 and h_la=0.001350.

As seen from the six plots in Figure 2, with the increasing number of TOP-N areas, the identification accuracy increases first, then stabilizes, and eventually tends to decrease. This is due to the fact that the number of frequently distributed areas of an object’s trajectory is limited. When TOP-N grows to a certain value and continues to increase, infrequently distributed areas are introduced. These infrequently distributed regions have weak regularity and do not exhibit periodicity. Therefore, the larger TOP-N will not only fail to improve identification accuracy, but also even reduce it. In addition, the TOP-N-COS method is significantly superior over the TOP-N-PRO method because the recognition accuracy of the TOP-N-PRO method is related to the probability of the user appearing in a given area. Moreover, it is affected by the method of dealing with the probability deviation of two trajectories appearing in non-co-distributed regions.

#### 4.3.3. Effects of Time Length

To further explore the effect of trajectory time length on recognition accuracy, we performed three sets of experiments on the GeoLife dataset, setting the length of time to 10 days, 30 days, and 50 days, respectively. For experimental comparisons, we retained 54 trajectories in the GeoLife dataset that are longer than 50 days in length to ensure that the trajectories of each set are from the same users. We follow the same experimental method as the previous section where each trajectory is divided into two segments. According to the experimental conclusions of Section 4.3.1 and Section 4.3.2, and considering the compromise between the amount of calculation and the identification accuracy, we set the h_lo=0.000578, h_la=0.00045 and TOP-N = 39.

The experimental results are shown in Table 1. We can find that the identification accuracy of the three methods substantially decreases with the shortening of the time length of the trajectory. When the trajectory time length is 50 days, the accuracy exceeds 80%. When the time length is reduced to 10 days, four methods are all reduced by about 20% in identification accuracy. This fact shows that the length of the trajectory has a great influence on the identification performance. Meanwhile, although the trajectory time is only 10 days, there is also a considerable possibility for successful identification.

#### 4.3.4. Comparisons with Other Algorithms

To validate user identification, we performed comparisons of our method with previous representative methods. Since our approach is designed for asynchronous scenarios, many existing identification algorithms are not applicable. For example, Kondor et al. proposed to achieve identification by time-space matching trajectory points [21]. It requires that the points of two trajectories are close in both time and space. Therefore, we compare our method with three feasible existing algorithms.

Hao et al. [22] proposed a cross-domain trajectory similarity method (UNICORN) based on TF-IDF (term frequency-inverse document frequency). TF-IDF transforms the original user trajectory into grid representation, where each cell can be treated as a word, and each user trajectory is seen as a paragraph. Each user trajectory is transformed into a vector by using the TF-IDF method to obtain trajectory similarity.

Zhang et al. [23] presented a very concise and effective method, called hot-matrix (HOT-MATRIX). HOT-MATRIX method produces hotspot matrix by meshing the map and counting the frequency of the trajectory appearing in each grid. Then the hotspot matrix is used to represent trajectory similarity.

Automatic user identification (AUI)presented in [24] matches the users by co-filtering the ID pairs with signal base similarity(SIG) and weighted Jaccard similarity (WJS).

In [27], a cross-domain trajectory matching algorithm based on paragraph2vec (CDTraj2vec) is presented. The method maps the trajectory into the grid, treats each small grid as a word and each trajectory as a paragraph, and then uses the PV-DM model in the paragraph2vec algorithm to extract the positional access order features in the trajectory sequence to obtain the vector representation of user trajectory. Finally, the similarity of the vector is used to represent the trajectory similarity.

We then show the performance evaluation of our TOP-N method. In order to find the best combination of parameters to reduce the amount of calculation and improve the recognition accuracy, we set the parameters TOP-N, h_lo, and h_la to 5–100, 0–0.006, 0–0.005, and did a lot of experiments. Finally, the parameter settings for datasets $D_{g_{1}}$ vs. $D_{g_{2}}$ and $D_{c_{1}}$ vs. $D_{c_{2}}$ are shown in Table 2 and Table 3, respectively.

As seen from Table 4, all methods have better performance on dataset $D_{g_{1}}$ vs. $D_{g_{2}}$ than $D_{c_{1}}$ vs. $D_{c_{2}}$. This is due to the difference of information recorded in the two datasets. First, many trajectories in GeoLife dataset used for experiments are more than 50 days in time span, and most of the time intervals between the adjacent points are about 5 s. In contrast, the time span of Cabspotting dataset is only 23 days, and the points are sparse. Second, GeoLife dataset records the wide-ranging outdoor activities of the tested users, including not only routines for going home and going to work, but also some entertainment and sports activities such as shopping, sightseeing, dining, hiking and cycling. Instead, because Cabspotting dataset is collected by devices embedded in taxis, the routes and trajectories are spatially limited to street, thus lacking private access. Therefore, the points in the Cabspotting dataset are not so discriminative, thus making identification more challenging. For the above reasons, these two datasets result in very different identification accuracy in our experiments.

From the experimental results, we can see the advantages of the TOP-N method in the asynchronous identification resolution provided that all three TOP-N variants outperform other comparison methods. HOT-MATRIX method achieves a relatively high accuracy while the UNICORN method and the AUI method obtain relatively poor identification results. For the UNICORN method, only the co-distributed regions are considered, but the influence of the non-co-distribution areas is ignored. When the trajectories of different users are sufficiently similar, it is difficult to distinguish them. For the CDTraj2vec method, although it can extract the order features of the location point access of the trajectory, it ignores the frequency of the visiting locations. The premise that the AUI method and CDTraj2vec method can identify users is that the employed dataset contains many stay points. However, the Cabspotting dataset contains very few stop points. Therefore, AUI and CDTraj2vec are not applicable to Cabspotting dataset that contains almost no stay points.

At last, as far as the computational cost is concerned, we give a brief discussion. HOT-MATRIX method needs a very huge computation because it uses cosine of the hot-matrix of two trajectories to express the similarity. UNICORN calculates cosine of all pairs in the same regions with a inverted-index strategy to reduce the computational cost. Although the computational complexity can be considerably reduced, it needs to find all co-distribution regions from a large number of trajectories. In our method, we only consider TOP-N frequently distributed regions in calculation similarity, which thus leads to reduced computational complexity. Experimentally, the TOP-N value corresponding to the highest identification accuracy stably falls between 30 and 100.

## 5. Conclusions

This paper explores the user identification resolution based on asynchronous trajectory information. The proposed method considers TOP-N most frequently distributed regions and exploit three applicable methods to measure trajectory similarity, including angle cosine, probability deviation and weighted Jaccard similarity. We validated the effectiveness of our algorithm in two real datasets. The experimental results shows that only the TOP-N most frequently distributed regions need to be compared for user identification task. Our method enjoys the highest computational efficiency because only TOP-N regions are used to compute trajectory similarity. Although our method shows good results, in the real application, since the number of frequently distributed areas (habitual access areas) varies for different users, the parameter TOP-N in the algorithm is not well determined. Consequently, in order to achieve higher identification accuracy, the values of h_lo and h_la should be small, so that when the user trajectory is in large range, such as across cities, the calculation will accordingly expand. In addition, the method does not consider the timing of the trajectory. How to consider the spatial stratification heterogeneity of the trajectory information is a challenging problem.

## Figures and Tables

**Table 1:** Experimental results on different time lengths on GeoLife dataset.

Time Spans	TOP-N-PRO	TOP-N-COS	TOP-N-WJS
50 days	0.8796	0.8333	0.8889
30 days	0.7639	0.8056	0.8287
10 days	0.6296	0.6265	0.6636

**Table 2:** Parameter Setting for $D_{g_{1}}$ vs. $D_{g_{2}}$.

Parameters	TOP-N-PRO	TOP-N-COS	TOP-N-WJS
h_lo	0.000578	0.002359	0.003538
h_la	0.00045	0.0018	0.0027
TOP−N	63	23	25

**Table 3:** Parameter setting for $D_{c_{1}}$ vs. $D_{c_{2}}$.

Parameters	TOP-N-PRO	TOP-N-COS	TOP-N-WJS
h_lo	0.000578	0.000578	0.000578
h_la	0.00045	0.00045	0.00045
TOP−N	69	69	99

**Table 4:** Comparison of results of different methods on two datasets.

Datasets	HOT-MATRIX	UNICORN	AUI	CDTraj2vec	TOP-N-PRO	TOP-N-COS	TOP-N-WJS
$D_{g_{1}}$ vs. $D_{g_{2}}$	0.5732	0.3537	0.3682	0.4421	0.5915	0.5854	0.6616
$D_{c_{1}}$ vs. $D_{c_{2}}$	0.3713	0.2397	∖	∖	0.4076	0.4086	0.4748
